# Study Protocol for the Japan Pregnancy, Eating, Activity, Cohort (J-PEACH) Study: Investigating Perinatal Maternal Lifestyle and Infant Health

**DOI:** 10.3390/mps8060128

**Published:** 2025-11-01

**Authors:** Megumi Haruna, Megumi Fujita, Masayo Matsuzaki, Mie Shiraishi, Naoko Hikita, Yoshiko Suetsugu, Yoko Sato, Kaori Yonezawa, Moeko Tanaka, Riko Ohori, Satoko Aoyama, Moeri Yokoyama, Ayano Takeuchi, Takeshi Nagamatsu, Satoshi Sasaki

**Affiliations:** 1 Department of Midwifery and Women’s Health, Division of Health Sciences and Nursing, Graduate School of Medicine, The University of Tokyo, Tokyo 113-0033, Japan; matsuzaki.masayo@tmd.ac.jp (M.M.); mi-shi@sahs.med.osaka-u.ac.jp (M.S.); hikita.naoko.419@m.kyushu-u.ac.jp (N.H.); kaoriyone@m.u-tokyo.ac.jp (K.Y.); tanaka-moeko48526@g.ecc.u-tokyo.ac.jp (M.T.); riko.soyogi@gmail.com (R.O.); aoyama-satoko125@g.ecc.u-tokyo.ac.jp (S.A.); moeri-yokoyama@g.ecc.u-tokyo.ac.jp (M.Y.); 2 Global Nursing Research Center, Graduate School of Medicine, The University of Tokyo, Tokyo 113-0033, Japan; 3 Department of Clinical Nursing, Graduate School of Medical Science, Yamagata University, Yamagata 990-9585, Japan; f.megumi@med.id.yamagata-u.ac.jp; 4 Department of Reproductive Health Nursing, Graduate School of Health Care Sciences, Institute of Science, Tokyo 113-8519, Japan; 5 Department of Children’s and Women’s Health, Graduate School of Medicine, The University of Osaka, Osaka 565-0871, Japan; 6 Department of Health Sciences, Graduate School of Medical Sciences, Kyushu University, Fukuoka 812-8582, Japan; suetsugu.yoshiko.742@m.kyushu-u.ac.jp (Y.S.); satou.youko.350@m.kyushu-u.ac.jp (Y.S.); 7 Department of Integrated Science and Engineering for Sustainable Societies, Faculty of Science and Engineering, Chuo University, Tokyo 112-8551, Japan; atakeuchi883@g.chuo-u.ac.jp; 8 Faculty of Medicine, Obstetrics and Gynecology, International University of Healthcare and Welfare, Chiba 286-8686, Japan; tnag-tky@iuhw.ac.jp; 9 Department of Social and Preventive Epidemiology, School of Public Health/Division of Health Sciences and Nursing, Graduate School of Medicine, The University of Tokyo, Tokyo 113-0033, Japan; stssasak@m.u-tokyo.ac.jp

**Keywords:** birth weight, gestational weight gain, healthy lifestyle, pregnancy, postpartum period

## Abstract

The prevalence of low-birth-weight infants has increased over the past 40 years to approximately 9–10% of Japanese live births. This study aims to identify healthy lifestyle behaviors and psychosocial factors contributing to appropriate perinatal outcomes, gestational weight gain, and postpartum weight change. The Japan Pregnancy, Eating, Activity, and Cohort study was initiated in 2020 in Tokyo, Yamagata/Miyagi, Osaka, and Fukuoka. Participants will be enrolled at approximately 12 weeks of gestation, with follow-up at 18–27 and 35–41 weeks of gestation and 1, 6, and 12 months postpartum. Approximately 3000 participants are targeted: Yamagata/Miyagi (n = 300), Tokyo (n = 1500), Osaka (n = 800), and Fukuoka (n = 400). Participants will complete questionnaires on healthy lifestyle behaviors (dietary intake, physical activity, and circadian rhythm), psychosocial factors, weight control, and behavioral intentions. Medical records will be reviewed for antenatal checkup data. The primary outcomes will include gestational weight gain, infant birth weight, perinatal complications, breastfeeding, and postpartum weight change. Demographic and psychosocial factors and lifestyle behaviors will be examined as covariates and potential confounders. Biological samples will be collected in Tokyo and Yamagata. The study’s findings will inform efforts to improve perinatal care guidelines through evidence-based recommendations.

## 1. Introduction

The prevalence of low-birth-weight (LBW) infants (<2500 g) has increased over the past 40 years to approximately 9–10% of live births in Japan, although Japan has one of the lowest perinatal mortality rates worldwide [1,2,3]. For 2023, the proportion of LBW infants to live births was reported to be 9.6%: 8.5% for boys and 10.8% for girls [1]. This rate is above the average for Organisation for Economic Co-operation and Development (OECD) countries (6.6%). Moreover, it is approximately double the average for Nordic and Baltic countries (4%) [4]. Numerous epidemiological studies, including large birth registries and cohorts, have suggested a link between LBW and chronic adult diseases such as Type II diabetes mellitus, hypertension, coronary artery disease, cancer, and various psychiatric disorders [5]. These findings can be explained by the Developmental Origins of Health and Disease hypothesis [6,7], which proposes that environmental conditions during the intrauterine and early life stages can be influenced by maternal malnutrition, severe stress, or disease and have long-lasting effects on health and disease risk in later childhood and adulthood. The underlying epigenetic mechanisms may play a role in long-term health effects [6,7]. Indeed, women with a low pre-pregnancy body mass index (BMI) [8] and low total gestational weight gain (GWG) have higher risks of delivering infants with LBW [9]. Although LBW is a surrogate marker of adverse fetal environmental conditions, the mechanisms underlying the relationship between higher risks of inadequate maternal body weight and birth weight remain unclear.

In Japan, the proportion of underweight (BMI < 18.5 kg/m^2^) and obese women (BMI ≥ 25 kg/m^2^) in their 20 s is 20.7% and 8.9%, respectively, and the respective proportions for women in their 30 s are 16.4% and 15.0% [10]. The similar rates of underweight and obesity among women in their 30 s highlight the importance of appropriate weight management for pregnant women with obesity [10].

Moreover, in Japan, 30.0% of new mothers are 35 years of age or older, leading to an increased risk of hypertensive disorders during pregnancy and gestational diabetes due to advanced maternal age [11]. To reduce perinatal risks, it is crucial to maintain optimal weight gain during pregnancy, especially for women who are underweight or obese before becoming pregnant.

Guidelines for GWG based on pre-pregnancy BMI have been developed to optimize maternal and fetal health outcomes by preventing both insufficient and excessive weight gain during pregnancy. In Japan, GWG recommendations, which, without robust evidence, initially emphasized weight restriction to manage hypertensive disorders, have evolved in recent decades [12]. In 2006, the Japanese Ministry of Health, Labour and Welfare (“the Ministry”) published new guidelines allowing a 7–12 kg weight gain for women with a normal BMI [13]. These guidelines differed from the Institute of Medicine guidelines recommending 11.5–16.0 kg for the same group [14]. In 2021, both the Japan Society of Obstetrics and Gynecology [15] and the Ministry [16] revised their guidelines, offering reference GWG values without specific recommendations due to limited evidence. Despite these efforts, more than half of pregnant Japanese women do not achieve the recommended GWG [17]. This issue resonates with global health priorities emphasized by the World Health Organization and aligns with Sustainable Development Goal (SDG) 3, which targets improved maternal nutrition and reduced perinatal risks. Addressing LBW and inadequate GWG through evidence-based guidance is essential for maternal and child health equity in Japan and across Asia, where region-specific data are needed.

Psychosocial factors are vital in providing appropriate health guidance. However, the impact of factors such as maternal age, education, employment, mental health, and health behaviors, such as diet, physical activity, sleep, and lifestyle, on GWG remains unclear. Few studies have investigated the health behaviors and changes necessary for effective weight control in clinical settings. Notably, the health behaviors and weight perceptions of underweight women are not well understood.

During early pregnancy, maternal body fat accumulates due to hyperphagia and increased lipogenesis. In contrast, fat deposits break down during late pregnancy to support fetal growth [18]. As metabolic changes occur throughout pregnancy, and the significance of maternal weight gain in each trimester varies, it is important to examine the relationship between maternal weight gain and pregnancy complications or fetal growth at each stage and throughout pregnancy. When analyzing repeated measurements such as maternal weight gain in each trimester of pregnancy, researchers should consider psychosocial factors and determine the specific points at which they affect pregnancy and childbirth outcomes.

Several large-scale birth cohort studies have been conducted in Japan, including the National Survey on Japan Environment and Children’s Study led by the Ministry of the Environment [19]. These cohort studies indicate that GWG is associated with LBW and/or macrosomia [20] and provide GWG growth charts to align with new guidelines [21]. However, more evidence is needed to understand how psychosocial factors and individual health behaviors, such as eating habits, physical activity, daily routines, target weight, and weight control, affect weight gain during each trimester of pregnancy. This knowledge gap hinders the provision of comprehensive health guidance.

Postpartum weight change also has implications for women’s preconception health in subsequent pregnancies, as well as during the premenopausal and postmenopausal stages [22]. However, few studies have examined the associations between lifestyle, eating behaviors, psychosocial factors, weight changes, and associated factors in postpartum women [23]. In fact, postpartum weight retention is associated with pre-pregnancy BMI, weight gain during pregnancy, and breastfeeding [24]. To address these gaps, our project will explore weight changes up to one year postpartum by considering factors such as nutritional intake, physical activity, lifestyle patterns, breastfeeding, employment, mental health, and weight control intention. This study aims to gather evidence on appropriate postpartum weight management.

This study is intended to clarify the relationship between maternal weight changes and psychosocial factors from early pregnancy to one year postpartum. By integrating longitudinal data and behavioral insights, it addresses key evidence gaps in Japan and Asia and contributes to the development of culturally appropriate, evidence-based health guidance aligned with global maternal and child health goals.

## 2. Methods and Design

### 2.1. Study Design and Setting

**Figure 1:**
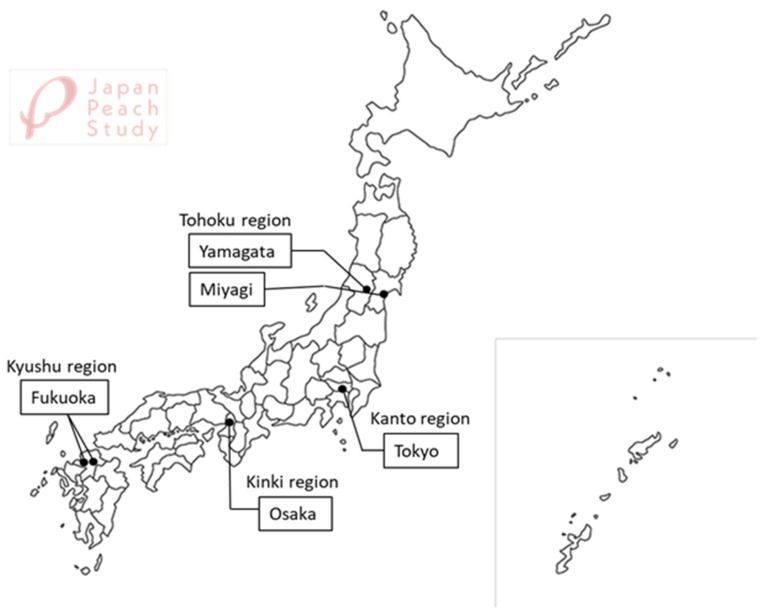
Study areas of the J-PEACH Study for 2020–2025.

The Japan Pregnancy, Eating, Activity, Cohort (J-PEACH) Study is a multicenter prospective cohort study that surveys women throughout pregnancy and childbirth, and up to one year postpartum in Japan. Midwifery researchers from four national universities (The University of Tokyo, Yamagata University, Osaka University, and Kyushu University) are conducting the cohort study using a unified protocol at each site and are collaborating on data linkage. The study covers four regions: the Tokyo metropolitan area (Kanto region: the southeastern part of Japan’s main island, Honshu), Yamagata and Miyagi Prefectures (Tohoku region: northeastern Honshu), Osaka metropolitan area (Kinki region: west central Honshu), and Fukuoka Prefecture (Kyushu region: the southernmost of the four major islands) (Figure 1). Data are being collected at six hospitals: the University of Tokyo Hospital in the Tokyo metropolitan area, two hospitals in the Tohoku region (Yamagata University Hospital in Yamagata Prefecture and a community-based acute care hospital in Miyagi Prefecture), a maternal clinic in Osaka in the Kinki region, and two hospitals in the Kyushu region (Kyushu University Hospital and a primary care hospital in Fukuoka). The first cohort study was initiated at the University of Tokyo Hospital in February 2020, and the research team has recruited participants via a protocol that was modified because of the COVID-19 pandemic. All paper-based surveys were changed to online surveys to minimize face-to-face interactions. Other sites have been added since 2021, and the survey process is ongoing.

### 2.2. Inclusion and Exclusion Criteria

#### 2.2.1. Inclusion Criteria

Eligible participants are those who (1) plan to give birth at a hospital that serves as the study site, (2) are able to understand and complete a questionnaire in Japanese, and (3) are aged 18 years or older. Pregnant women with significant underlying health conditions or multiple pregnancies are also eligible.

#### 2.2.2. Exclusion Criteria

Women judged by clinical staff to be unavailable for research participation are excluded.

### 2.3. Recruitment and Consent

Midwifery researchers recruit potential study participants during face-to-face antenatal checkups. Participants are provided with the study information statements, and written informed consent is obtained.

Participants play a crucial role in this study. We have improved the survey process based on their comments. To encourage participation, we shared the individual results. Additionally, we provide plain-language research summaries on our website (https://j-peach.jpn.org/en/ accessed on 13 August 2025) to non-experts, including the participants themselves.

### 2.4. Study Protocol

**Figure 2:**
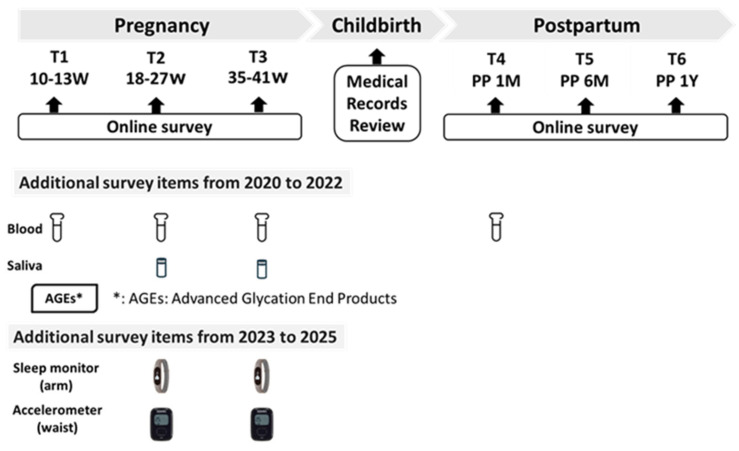
The measurement protocol of the J-PEACH Study for 2020–2025.

This multicenter prospective cohort study has been conducted since February 2020 and is ongoing. Prospective data are being collected from participants at four study sites via questionnaires, as outlined in Table 1, at three time points during pregnancy (10–13, 18–27, and 35–41 weeks) and at three time points after childbirth (1, 6, and 12 months postpartum). Blood samples are only collected from participants at the Tokyo site during pregnancy (10–13, 18–27, and 35–41 weeks) and one month postpartum. Saliva samples are collected only from Yamagata during pregnancy (18–27 and 35–41 weeks) (Figure 2). Therefore, analyses will include site as a covariate, and stratified sensitivity analyses will be conducted to ensure appropriate interpretation based on regional sampling characteristics.

### 2.5. Primary Outcomes and Potential Determinant Factors (Figure 3)

This study focuses on three primary outcomes: GWG, infant birth weight, and maternal and neonatal health. Specifically, we aim to investigate the following factors:

(1) Current status of maternal lifestyle during pregnancy and its impact on GWG;
(2) Potential determinant factors of infant LBW;
(3) Potential determinant factors of maternal and neonatal health outcomes.

The target health outcomes include preterm birth, gestational diabetes mellitus, hypertensive disorders of pregnancy, anemia, and perinatal depression. Variables related to maternal lifestyle during pregnancy include dietary intake, physical activity, sleep, circadian rhythms, weight control, intention to control weight, and weight-control behavior.

**Figure 3:**
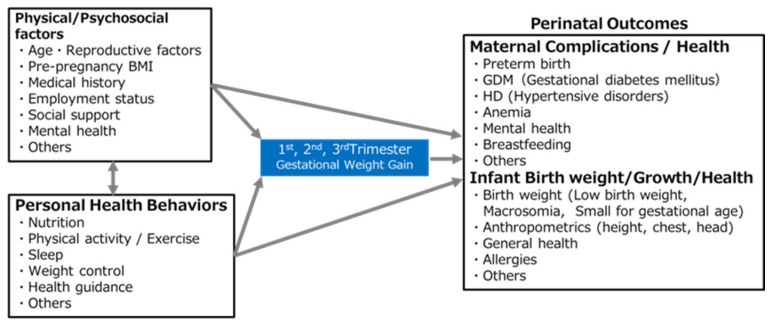
Conceptual framework of factors influencing perinatal outcomes.

### 2.6. Secondary Outcomes

In addition to the primary outcomes, the study includes secondary outcomes, including delivery mode, postpartum weight change, and breastfeeding.

### 2.7. Measures (Scales)

#### 2.7.1. Anthropometric Measurements

Pre-pregnancy weight and height are self-reported, and significant differences in weight are assessed by comparing the pre-pregnancy and early pregnancy values. Maternal gestational and infant weights are obtained from health checkup records. Body weight is measured using calibrated digital scales, maintained to Japan’s Measurement Act and hospital protocols.

#### 2.7.2. Dietary Intakes and Behaviors

Dietary intake is assessed using the Brief Self-Administered Diet History Questionnaire (BDHQ), a fixed-portion, 58-item questionnaire. The BDHQ was developed to assess the habitual intake of foods and more than 100 nutrients, and dietary behaviors at the individual level [25,26]. The validation of pregnant Japanese women has been confirmed using blood and 24-h urine samples [27].

#### 2.7.3. Physical Activity

The Physical Activity in Pregnancy Questionnaire (PPAQ) was originally developed in the United States and is widely used to assess and measure physical activity levels among pregnant women [28,29]. The Japanese version of the PPAQ includes 33 activities categorized as follows: household/caregiving (13 activities), occupational (5 activities), sports/exercise (8 activities), mobility (4 activities), and inactivity (3 activities) [30]. Respondents select the category that best represents the amount of time spent on each activity in the previous month and add any additional activities that were not listed. Results are calculated for each physical activity intensity categorized by metabolic equivalents (METs) (Sedentary: 1–1.5 METs; Light: >1.5–<3.0 METs; Moderate: 3.0–<6.0 METs; Vigorous: ≥6.0 METs) and for each activity type. Additionally, “smartphone use” has been added to the “watching TV or a video” section to better accommodate current lifestyles.

#### 2.7.4. Mental Health

Antenatal and postnatal depressive symptoms are assessed using the Edinburgh Postnatal Depression Scale (EPDS). The EPDS was developed by Cox et al. (1987) [31] to screen for postpartum depression. It consists of 10 questions scored from 0 to 3, with a total score of 0–30. A previous meta-analysis using individual participant data found that an EPDS cutoff score of 10/11 provides the best balance between sensitivity and specificity. A cutoff score of 12/13 is less sensitive but more specific. To identify pregnant and postpartum women with higher symptom levels, a cutoff score of 12/13 can be used. However, lower cutoff scores should be used to avoid false negatives and identify most patients who meet the diagnostic criteria [32]. The Japanese version of the EPDS has been validated for use in the first month postpartum, and the cutoff point is set at 8/9 points [33]. Based on a Japanese study, we have selected 12/13 as the optimal EPDS cutoff score in the second trimester [34]. A previous prospective cohort study demonstrated a stable factor structure for the EPDS throughout the peripartum period [35].

#### 2.7.5. Severity of Nausea and Vomiting

A modified Pregnancy-Unique Quantification of Emesis and Nausea (modified-PUQE) scale, a validated index, is used to assess the severity of nausea and vomiting during the first trimester [36]. The original version, the Motherisk Pregnancy Quantified Emesis (PUQE) Index, was designed to measure the three physical symptoms (nausea, vomiting, and retching) over the previous 12 h [37]. The Japanese version of the modified PUQE is based on the Japanese version of the PUQE-24, which has been validated in pregnant Japanese women during the first trimester [38]. In this study, the severity of nausea and vomiting during pregnancy is assessed as a subjective symptom over the previous month to align with the timing of other survey items. Severity is evaluated based on the total scores: (a) mild (3–6), (b) moderate (7–12), and (c) severe (≥13).

#### 2.7.6. Woman Abuse Screening

It is crucial to explore the mechanisms through which violence against women affects birth weight. While a previous systematic review suggested that abuse during pregnancy might be a modifiable risk factor for LBW [39], further research is needed to understand the influence of social factors on physiology and pregnancy outcomes. The short version of the Woman Abuse Screening Tool (WAST-Short), a two-item questionnaire, was developed to detect intimate partner violence [40], based on the 10-item original version of the WAST [41]. The WAST-Short is a valid tool for assessing non-physical violence, such as psychological abuse and relationships involving power and control. The validity of the Japanese version of the WAST-Short has been confirmed in a perinatal health setting. The sensitivity and specificity are 71.4% and 89.7%, respectively. It demonstrates good accuracy via alternate cutoff points, with a suggested high risk of ≤3 (which indicates responses of “some tension” or “some difficulty” [2 points] on one item and “much tension” or “great difficulty” [1 point] on the other) [42].

#### 2.7.7. Sense of Coherence

Sense of coherence (SOC) among pregnant women is linked to their postpartum perception of well-being [43]. SOC reflects one’s capacity to handle stress and lies at the core of the salutogenesis theory. SOC comprises three underlying elements: manageability, meaningfulness, and comprehensibility. Antonovsky [44], an SOC advocate, created two SOC scales: a comprehensive 29-item version (SOC-29) and a shorter 13-item version (SOC-13). A newly devised version of the University of Tokyo Health Sociology SOC Scale (SOC-3-UTHS) was developed for population surveys and has demonstrated significant correlation with the SOC-13 scale. It has shown satisfactory convergent and concurrent validities [45]. In this study, participants complete the SOC-3-UTHS and rate “manageability,” “meaningfulness,” and “comprehensibility” on a 7-point scale.

#### 2.7.8. Subjective Sleep Quality

The Pittsburgh Sleep Quality Index (PSQI) is a standardized questionnaire developed by the Department of Psychiatry at the University of Pittsburgh to measure sleep quality [46] and is used in clinical settings and research on sleep disorders. The Japanese version (PSQI-J) was developed by Doi et al. in 1998 and has demonstrated sufficient discriminatory power, with sensitivity and specificity of over 80% [47]. Seven component scores (C1: sleep quality, C2: sleep onset time, C3: sleep duration, C4: sleep efficiency, C5: sleep difficulty, C6: use of sleeping pills, and C7: difficulty staying awake during the day) are calculated based on 19 self-assessment items, and the total score is used to determine overall sleep quality. Each component is scored from 0 to 3 points, and the total score is calculated from 0 to 21 points. Generally, a score of 5.5 is considered normal, with scores of 5 or less indicating no sleep problems and scores of 6 or higher indicating suspected sleep disorders [47]. It is also useful in studies of pregnant women [48].

#### 2.7.9. Sleepiness Scale

The Epworth Sleepiness Scale (ESS) was developed as a method for measuring the degree of daytime sleepiness. It correlates highly with the severity of respiratory disorders and blood oxygen saturation in patients with obstructive sleep apnea [49]. The Japanese version of the ESS questionnaire has been standardized, and both its reliability and validity have been verified [50]. Participants rate the likelihood of falling asleep on a four-point scale from 0 (almost none) to 3 (very high) for eight situations in daily life that may cause sleepiness. A total score of 24 points is calculated, with a higher score indicating greater sleepiness.

### 2.8. Measures (Devices)

#### 2.8.1. Accelerometer

Physical activity levels in the participants’ daily lives are measured using Active Style Pro HJA-750C (Omron Healthcare Co., Ltd., Kyoto, Japan). This is a three-axis accelerometer that is worn around the waist. The measured data are processed using macros provided free of charge by the Physical Activity Research Platform [51], and variables such as total number of steps (steps) per day, physical activity time (minutes) divided into daily activities and walking according to activity intensity, and total physical activity (METs-hours) are extracted.

#### 2.8.2. Sleep Monitor

Objective sleep is measured using either the Axivity AX3 (Axivity Ltd., Newcastle-upon-Tyne, UK) or ACCEL POLARIS (ACCELStars, Inc., Tokyo, Japan) device loaned by ACCELStars, Inc., a joint research institution [52]. Axivity is a three-axis accelerometer that has been used in various studies, including the UK Biobank study [53,54]. This device measures 23 × 32.5 × 7.6 mm and weighs only 11 g, making it small and lightweight. It is non-invasive and can be worn without causing significant discomfort to the wearer. ACCEL POLARIS is a sleep monitor developed by ACCELStars, Inc., that measures 38 × 52 × 17 mm, weighs 45 g, and can also measure pulse waves for wear detection. Both devices use the ACCEL algorithm [54,55], developed by the research group of co-researcher Hiroki R. Ueda, to identify and analyze 24-h sleep/wake rhythms based on the movement of accelerometers on the arms. The ACCEL algorithm is highly accurate, with a specificity of 82.2% when sleep and wakefulness data from polysomnography tests are used as the gold standard, and an accuracy of 93.2% and sensitivity of 97.2% [54,55].

### 2.9. Sample Size

This study is primarily intended to identify the factors related to newborn birth weight. A previous systematic review and meta-analysis reported an increased risk of LBW infants in cohort studies among underweight women in developed countries, with an adjusted relative risk (RR) of 1.48 (95% confidence interval [CI]: 1.29–1.68) [56]. Because 9.4% of all births in Japan are LBW, we estimate that the risk of an underweight woman giving birth to an LBW infant is approximately 13.9%. The sample size has been calculated using the G*Power 3.1 statistical power analysis program with the following parameters: a two-tailed test, proportions (proportion of exposed to the outcome of interest: p1 = 0.139 and proportion of unexposed to the outcome of interest: p2 = 0.094), alpha error probability of 0.05, power of 0.80, and an allocation ratio of 4. This yields the required sample sizes of 2460 and 2952, respectively, which account for a 20% dropout rate. Therefore, the target enrolment number is set at approximately 3000. Participants are allocated proportionally based on the number of deliveries in 2019 across the four research areas: Yamagata/Miyagi (n = 300), Tokyo (n = 1500), Osaka (n = 800), and Fukuoka (n = 400).

### 2.10. Statistical Analysis

Descriptive statistics are used to present the participants’ profiles and summarize the exposure and outcome variables. Continuous variables are reported as means, standard deviations, medians, and ranges. Group comparisons for continuous variables are conducted using Student’s *t*-tests or analysis of variance (ANOVA), whereas the Mann–Whitney U test or a Kruskal–Wallis test is used if the assumptions for Student’s *t*-tests or ANOVA are not met. Categorical variables are presented as percentages and frequencies, and group comparisons are performed using chi-squared (χ^2^) tests. Initially, univariate models are used to examine the relationship between exposure variables and each outcome. The interaction terms between the independent variables are examined to identify potential effect modifications. Adjustments are made for potential confounders in the multivariate mixed-effects models. Logistic and linear regression models report the crude and adjusted odds ratios (ORs), along with their associated 95% CIs and coefficients with 95% CIs, respectively.

A multilevel analysis is used to interpret the data as it considers the hierarchical structure and accounts for within-group dependence and variability between groups. This approach allows for the estimation of individual- and group-level effects and provides a more accurate analysis than traditional single-level models.

Self-reported weights are used only when clinical data are unavailable. Sensitivity analyses assess the impact of discrepancies between self-reported and measured values.

In the primary analysis, we assess the validity of key assumptions regarding random missing data and carefully consider suitable methods to manage it. In the sensitivity analysis, we employ diverse assignment methods and investigate the potential influence of the missing data on the outcomes. We compare the participants’ characteristics with missing data between the two groups, namely, those with favorable outcomes and those with unfavorable outcomes. In addition to analyzing gestational weight gain and postpartum weight change as outcomes, we use trajectory analysis to determine the patterns of weight change. Statistical analyses are performed using the SPSS (version 28.0^®^; IBM Japan Inc., Tokyo, Japan) and SAS statistical software (version 9.4; SAS Institute Inc., Cary, NC, USA).

### 2.11. Dissemination of Study Findings

The research findings will be published in peer-reviewed scientific journals, and a plain-language summary will be made available. The results will be presented at national and international conferences. These findings are expected to contribute to the improvement of perinatal care guidelines.

## 3. Results

Recruitment for the J-PEACH study began in February 2020 and remains ongoing. The first dataset was temporarily finalized in June 2024, and the second in April 2025. By April 2025, 2108 participants had been enrolled, representing approximately 70.3% of the target sample size of 3000. A summary of the study participants is shown in Table 2.

## 4. Discussion

This multicenter prospective cohort study is designed to inform maternal health guidance by collecting comprehensive data from perinatal women. The collaborative research team consists of midwifery researchers, nutritional epidemiologists, statisticians, obstetricians, and graduate students specializing in midwifery, working together to address the clinical questions raised by midwives involved in maternal health guidance in clinical settings. Comprehensive data have been collected on various aspects of perinatal women’s lifestyle behaviors, including nutrition, exercise, physical activity, sleep, daily routines, weight management, and perinatal health. Women’s intentions regarding health behaviors and the type of health guidance they have received have also been examined. These validated tools will be used to assess dietary nutrition, physical activity, sleep, and mental health. Regular clinical chart reviews and systematic data storage will also be conducted.

The prevalence of LBW in this study was 13.8%, exceeding the national average of 9.6% reported in the 2023 Annual Report on Vital Statistics [1]. This may reflect the inclusion of university hospitals, which typically serve a higher proportion of high-risk pregnancies.

Several limitations should be considered when interpreting the findings. The study population was limited to Japanese-speaking women recruited from hospitals, and follow-up was conducted only up to one year postpartum. As regional and socioeconomic biases may be present within the study population, these factors will be considered in the analysis. They may affect the generalizability of the results and limit the assessment of long-term outcomes. Additionally, the length of the questionnaire may have contributed to participant burden and potential attrition.

Despite these limitations, the study has notable strengths. It includes rich data on psychosocial factors, health behaviors, received health care, and medical records. The inclusion of Tokyo—the region with the highest number of births in Japan—further enhances the relevance of the findings to national maternal health strategies. The J-PEACH study ultimately aims to provide evidence-based recommendations for maternal health behaviors that reduce the risk of adverse obstetrical outcomes and improve early-life health trajectories. J-PEACH is a newly established cohort that captures perinatal data from 2020 onward, providing timely insights into maternal and child health in a contemporary context. Its design complements existing Japanese birth cohorts by facilitating individual participant data integration and collaborative analyses. Through this approach, J-PEACH enables investigation of research questions that may be difficult to address within a single cohort, thereby enhancing both the generalizability and analytical depth of findings relevant to current perinatal care.

## Figures and Tables

**Table 1:** Questionnaire items, clinical medical record data, measurements, and samples.

	Pregnancy			Childbirth	Postpartum		
	T1	T2	T3		T4	T5	T6
	10–13 Weeks	18–27 Weeks	35–41 Weeks		1 Month	6 Months	12 Months
**Questionnaire items**							
m-PUQE	✓	✓	✓				
BDHQ		✓	✓		✓	✓	✓
PPAQ		✓	✓		✓	✓	✓
EPDS		✓	✓		✓	✓	✓
WAST-short		✓	✓		✓	✓	✓
SOC-3		✓	✓		✓	✓	✓
PSQI		✓	✓		✓	✓	✓
ESS		✓	✓		✓	✓	✓
**Demographics** **and lifestyle factors**							
**<Mother>**							
Age	✓	✓		✓			
Educational attainment		✓					
Employment		✓					
Marital status		✓	✓				
Alcohol consumption		✓	✓				
Smoking status		✓	✓				
Family member		✓	✓				
Medication		✓	✓				
Supplement intake		✓	✓				
Lifetime habits (sleep, meals, sedentary)		✓	✓				
Meal skipping		✓	✓		✓	✓	✓
Night-time fasting period		✓	✓		✓	✓	✓
Meal frequency		✓	✓		✓	✓	✓
Subjective general health		✓	✓				
Weight control, self-efficacy		✓	✓				
Body image		✓	✓				
Health guidance, target weight		✓	✓				
Maternal feelings toward pregnancy and fetus		✓	✓				
Family support		✓	✓				
Maternal skin trouble		✓	✓				
Self-reporting maternal weight		✓	✓		✓	✓	✓
**<Infant>**							
Weight, Height, Chest, Head				✓	✓	✓	✓
Feeding type					✓	✓	✓
Breastfeeding status					✓	✓	✓
Complementary feeding							✓
General health, Allergies					✓	✓	✓
**Hospital medical records**							
Medical history		✓	✓	✓			
Infertility treatment history		✓					
Pregnancy complications		✓	✓	✓			
Glucose tolerance tests		✓	✓				
Maternal height		✓					
Maternal weight		✓	✓	✓	✓	✓	✓
Gestational weight gain		✓	✓	✓			
Blood pressure		✓	✓	✓	✓		
Mode of delivery				✓			
**Measurements/samples**							
Sleep monitor	✓	✓	✓				
Accelerometer	✓	✓	✓				
AGEs *	✓						
Blood sample *	✓	✓	✓		✓		
Saliva sample **		✓	✓				

Note: * Only at Tokyo site, ** Only at Yamagata site. m-PUQE, modified Pregnancy-Unique Quantification of Emesis and Nausea questionnaire; BDHQ, Brief Self-Administered Diet History Questionnaire; PPAQ, Pregnancy Physical Activity Questionnaire; EPDS, Edinburgh Postnatal Depression Scale; WAST-short, Woman Abuse Screening Tool-Short; SOC-3, 3-item Sense of Coherence Scale; PSQI, Pittsburgh Sleep Quality Index; ESS, Epworth Sleepiness Scale. Sleep monitor: Axivity AX3 (Axivity Ltd., Newcastle-upon-Tyne, UK); ACCEL POLARIS (ACCELStars, Inc., Tokyo, Japan). Accelerometer: HJA-750C Active-style Pro (Omron Healthcare Co., Ltd., Kyoto, Japan). AGEs (Advanced Glycation End Products), measured using AGE Reader mu (DiagnOptics Technologies B.V., Groningen, The Netherlands).

**Table 2:** Number and characteristics of participants (2020–2025).

Variable	n (%) or Mean ± SD
**Total number of participants**	
Number of individuals recruited	2290
Participants who provided consent	2108
**Study regions**	
Yamagata/Miyagi	334 (15.8)
Tokyo	1027 (48.7)
Osaka	309 (14.7)
Fukuoka	438 (20.8)
**Maternal characteristics**	
Age at recruitment (years) (n = 2102)	33.9 ± 4.8
Height (cm) (n = 2087)	159.0 ± 5.5
Weight (kg) (n = 2085)	54.5 ± 9.6
Pre-pregnancy BMI (kg/m^2^) (n = 2085)	21.5 ± 3.6
BMI categories	
<18.5	312 (14.8)
≥18.5–<25	1502 (71.3)
≥25	271 (12.9)
Missing data	23 (1.1)
Parity status	
Primiparous women	1089 (51.7)
Multiparous women	1000 (47.4)
Missing data	19 (0.01)
Mode of delivery	
Vaginal birth	1339 (63.5)
Cesarean section	622 (29.5)
Missing data	147 (7.0)
Gestational age at birth	
Term birth	1948 (92.4)
Preterm birth	160 (7.6)
**Infant characteristics**	
Singleton vs. Multiple births (n = 2108)	
Singleton births	2026 (96.1)
Male infants	1010 (49.9)
Female infants	1016 (50.1)
Multiple births	79 (3.7)
Missing data	3 (0.1)
Birth weight (g) (n = 2031)	2943 ± 508
Low birth weight (<2500 g) (n = 2031)	281 (13.8)

Note: n, number of participants; SD, standard deviation; BMI, body mass index. Data collection is ongoing; data as of 14 April 2025. Missing data indicates unavailable responses at the time of analysis.

## Data Availability

The datasets generated during this study are not publicly available because of the restrictions imposed by the Ethics Committee. However, they can be obtained from the corresponding author upon request.

## Appendix A

Working members of the J-PEACH Study Group as of 2020–2025.

**The University of Tokyo, Tokyo, Japan:** Emi Tahara-Sasagawa, Yuriko Usui, Keiko Nakano, Nao Nishihara, Nana Wakabayashi, Tomomi Ohyama, Sakurako Kishino, Sarasa Habe, Momoko Kono, Mirei Kobayashi, Nana Ogawa, Reiha Onishi, Aoi Takayama, and Amane Koya.

**Yamagata University, Yamagata, Japan:** Momoka Yoshimura, Momoka Higuchi, Ai Shibata, Miharu Suzuki, Nao Saito, and Nanami Yasuno.

**Osaka University, Osaka, Japan:** Chisato Korogi, Miu Tsunekawa, Shoko Kowada, Sana Kato, Ayame Taniguchi, Natsuki Hori, Kaori Matsuda, and Riko Matsumoto.

**Kyushu University, Fukuoka, Japan:** Naho Miyazaki, Miyuki Obara, Aiko Tohara, Anna Shibata, Sakura Koga, Airi Fukuhara, Wakako Kozuma, and Aoi Baba.
